# Editorial on Asymptomatic Rib Fractures and Their Relationship to Child Abuse

**DOI:** 10.3390/children11101157

**Published:** 2024-09-24

**Authors:** Oliver J. Muensterer, Eva Möhler

**Affiliations:** 1 Department of Pediatric Surgery, Dr. von Hauner Children’s Hospital of the LMU University Hospital, 80377 Munich, Germany; 2 Department of Child and Adolescent Psychiatry, Saarland University Medical Center, 66421 Homburg, Germany; eva.moehler@uks.eu

*Children* recently published a Special Issue that focuses on child abuse and neglect as severe adverse childhood experiences. Multiple investigations have revealed a profound effect of child abuse or neglect on epigenetic aging and brain structural alterations as well as mental, physical, and social health throughout the lifespan, with traces still detectable even very late in life. Epidemiological data suggest global prevalence rates of up to 20% for childhood sexual abuse and even higher rates of up to 23% for childhood physical abuse. Furthermore, endocrine consequences, e.g., potentially altered HPA-axis functioning after a history of childhood abuse, has recently been described.

A growing body of research started to suggest that child abuse not only causes considerable and longstanding complications for the person concerned, but may provoke considerable transgenerational effects by acting as a risk factor for mental health in the offspring, e.g., through prenatal programming, signifying that not only victims of childhood abuse themselves are at risk for maladaptive development, but that childhood maltreatment appears to have a considerable negative impact on the next generation as well. The possibility that experiences of childhood abuse could interfere with later parenting behaviour, perceptions of an individual’s own parenting abilities, and perceptions of and interactions with the child underlines the utmost importance of prevention as a major public health goal with benefits extending further than one generation.

Several included articles have stressed psychosocial factors such as social support, poverty, or substance abuse when determining the risk and/or prevention of abuse. Another major advantage of this Special Issue is its multicultural content, providing insight into different approaches in multiple countries.

The majority of the articles in this issue focus on endeavors to prevent or interrupt child abuse, which, in light of the abovementioned facts, is of utmost scientific and clinical relevance and should be a priority for all mental health professions. On this note, and considering the highly relevant psychosocial aspects and impact, controversial viewpoints are a natural consequence and are also displayed here.

One of the most important aspects is the reliable detection of possible non-accidental trauma in children. This implies that clinicians maintain a high level of suspicion for certain typical physical findings and presentation history. Historically, posterior rib fractures have been considered virtually pathognomonic for child abuse.

Explicitly published as a “Hypothesis” in this Special Issue is an article by van Gemert et al. [1] that has raised substantial controversy. The authors present their view that some rib fractures may actually result from underlying and predisposing co-morbidities such as Vitamin D deficiency or osteogenesis imperfecta, and may, therefore, result in false accusations.

This topic is important enough to warrant further discussion because, on the one hand, it is essential to accurately detect child abuse in order to protect victims in a timely and effective manner. On the other hand, it is important to diagnose and treat the potential underlying issues in a developing child, and to exonerate caregivers who are not perpetrators.

Most experts in the field ascertain that the overwhelming majority of rib fractures, particularly posterolateral rib fractures, in infants and children are due to child abuse [2,3,4,5]. However, in select special situations, rib fractures may occur on the basis of premature osteopenia [6], Vitamin D deficiency [7], or osteogenesis imperfecta [8], although they are exceedingly rare, even after cardiopulmonary resuscitation [9]. If clinically indicated, predisposing co-morbidities should be ruled out.

The German association of child protection in medicine has commented specifically on the article by van Gemert et al. [1] In their comment [10], the authors criticize that the article by van Gemert et al. presents an untested hypothesis—that rib fractures might be even more likely to occur without non-accidental trauma—by indirectly estimating the possible incidences of alternative etiologies; this is an approach, that, according to the authors of the letter, cannot be regarded as a sound scientific assessment of evidence. In their comment targeting this article, Berthold et al. raise the concern that this publication is deliberately written to downplay the specific significance of posterior rib fractures, thereby reducing the detection of child abuse.

In this context, it is important to note that the manuscript was submitted and published merely as a hypothesis and not a scientific study. The authors acknowledge that the calculations are based on estimations and reports from other countries, because exact statistics of the incidence of rib fractures and their association with child abuse are unavailable in the Netherlands. Therefore, this article should be taken as intended—a hypothesis that remains to be tested and confirmed or rejected. The article is not intended to provide any sort of evidence and, therefore, under no circumstance should it be used or referenced in a court of law.

We do believe, however, that the discussion generated by this article shows that further research on the association between rib fractures, child abuse, and other less common underlying entities should be encouraged. Patterns typical of rib fractures resulting from child abuse and other rare entities should be identified. Artificial intelligence may be helpful in the diagnostic workup in the future.

In summary, the multiple endeavors to detect, prevent, or interrupt child abuse are an important and dominant focus of our Special Issue, validating the fact that this frequent phenomenon as a major public health threat should be a priority for all medical and mental health professions.
